# Differential expression of long non-coding RNAs under *Peste des petits ruminants virus* (PPRV) infection in goats

**DOI:** 10.1080/21505594.2022.2026564

**Published:** 2022-02-06

**Authors:** Aruna Pandey, Waseem Akram Malla, Amit Ranjan Sahu, Sajad Ahmad Wani, Raja Ishaq Nabi Khan, Shikha Saxena, P. W. Ramteke, Manas Ranjan Praharaj, Amit Kumar, Kaushal Kishor Rajak, Bina Mishra, D. Muthuchelvan, Basavaraj Sajjanar, Bishnu Prasad Mishra, Raj Kumar Singh, Ravi Kumar Gandham

**Affiliations:** a Division of Veterinary Biotechnology, ICAR-IVRI, Bareilly, India; b Department of Biological Sciences, SHUATS, Allahabad, India; c Genomics and Bioinformatics, National Institute of Animal Biotechnology, Hyderabad, India; d Division of Animal Genetics and Breeding, ICAR-IVRI, Bareilly, India; e Division of Biological Products, ICAR-IVRI, Bareilly, India; f Division of Virology, ICAR-IVRI, Nainital, India

**Keywords:** lncRNA, goat, immune response, PPR, RNA-Seq, PPRV

## Abstract

*Peste des petits ruminants* (PPR) characterized by fever, sore mouth, conjunctivitis, gastroenteritis, and pneumonia, is an acute, highly contagious viral disease of sheep and goats. The role of long non-coding RNAs (lncRNAs) in PPRV infection has not been explored to date. In this study, the transcriptome profiles of virulent Peste des petits ruminants virus (PPRV) infected goat tissues – lung and spleen were analyzed to identify the role of lncRNAs in PPRV infection. A total of 13,928 lncRNA transcripts were identified, out of which 170 were known lncRNAs. Intergenic lncRNAs (7625) formed the major chunk of the novel lncRNA transcripts. Differential expression analysis revealed that 15 lncRNAs (11 downregulated and 4 upregulated) in the PPRV infected spleen samples and 16 lncRNAs (13 downregulated and 3 upregulated) in PPRV infected lung samples were differentially expressed as compared to control. The differentially expressed lncRNAs (DElncRNAs) possibly regulate various immunological processes related to natural killer cell activation, antigen processing and presentation, and B cell activity, by regulating the expression of mRNAs through the cis- or trans-regulatory mechanism. Functional enrichment analysis of differentially expressed mRNAs (DEmRNAs) revealed enrichment of immune pathways and biological processes in concordance with the pathways in which correlated lncRNA-neighboring genes were enriched. The results suggest that a coordinated immune response is raised in both lung and spleen tissues of the goat through mRNA-lncRNA crosstalk.

## Introduction

*Peste des petits ruminants* (PPR) is one of the most prevalent infectious diseases among goats and sheep caused by the *Peste des petits ruminants virus* (PPRV). It is considered an emerging economically important disease. The etiologic agent PPRV is an enveloped, single-stranded negative-sense RNA virus that belongs to the Morbillivirus genus of the family Paramyxoviridae [1]. The disease affects the sheep and goat population across sub-Saharan Africa, Arabian Peninsula, and the Indian subcontinent [2]. Reports also suggest that goats are more susceptible to a severe form of the clinical signs and pathology than sheep [3–8].

lncRNAs are mRNA-like transcripts (longer than 200nt) but unlike mRNA, they do not encode proteins. They are transcribed by RNA Polymerase II, and are capped and spliced [9,10]. Evidence indicates that lncRNAs act as versatile molecules in regulating various biological processes, such as differentiation, apoptosis, and immune responses [11–13]. lncRNAs regulate gene expression by regulating transcriptional factors, inducing chromatin modification, affecting RNA processing events, sponging microRNAs (miRNAs), and affecting RNA stability [14]. While a substantially large number of studies have investigated lncRNAs in various types of cancers as therapeutic vulnerabilities and prognostic markers, the role that lncRNAs play to regulate innate as well as acquired immune processes has not been extensively studied in animals. Accumulating evidence supports the important role of host-encoded lncRNAs in regulating immune response [15–17]. Some of the recent examples include: lncRNA OASL-IT1 and interferon loop system that regulates antiviral defense against Zika Virus [18]; lncRNA NLRP3 promotes NLRP3-triggered inflammatory response in early acute lung injury by binding to miR-138-5p [19]; mRNA-lncRNA crosstalk is involved in immune regulation of carp macrophages [20]; lncRNA THUMPD3-AS1 is downregulated in osteoarthritis and when overexpressed, it promotes cell proliferation, reduces apoptosis and facilitates inflammatory response [19]. Besides, host-encoded lncRNAs have also been found to regulate viral infection. For example, the host-encoded lncRNA NRAV downregulates interferon-stimulated gene transcription [21] while NEAT1 lncRNA facilitates IL-8 production in response to influenza virus and herpes simplex virus infection [22].

In recent years, the RNA-Seq approach has facilitated the identification and annotation of lncRNAs [23–26]. By using RNA-Seq, viruses such as enterovirus, influenza virus, human immunodeficiency virus (HIV), hepatitis B and C viruses, and the SARS coronavirus have been shown to alter the expression of lncRNAs [27]. In our previous studies, RNA-Seq was applied to examine the role of transcription factors and miRNAs in regulating the immune responses under PPRV [28–31]. In the present study, we hypothesized that PPRV could regulate the expression of lncRNAs in infected goats, which in turn modulate how the host immune system responds to the viral infection. To evaluate this, RNA-Seq profiling of the lung and spleen tissues of PPRV infected goats and PPRV negative healthy goats was carried out. The results showed a tissue-specific dysregulation in lncRNA expression under PPRV infection. Functional analysis of these DElncRNAs revealed how lncRNAs regulate immune response under PPRV infection. Gene ontology analysis of differentially expressed mRNAs (DEmRNAs; known genes) also supports the potential role that lncRNAs play in modulating the host immune response. To our knowledge, this is the first study to report the lncRNA expression profile in PPRV infected goats.

## Results

### Confirmation of PPRV infection

**Figure 1.**
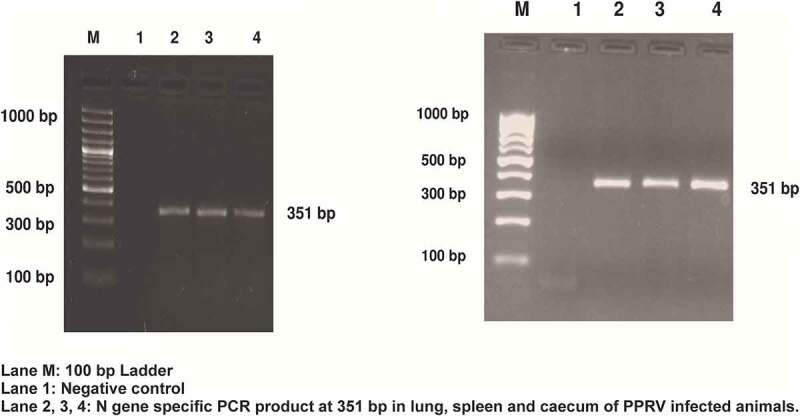
**Confirmation of PPRV infection in lung, spleen, and cecum tissue of goats** (a and b). Amplification of 351 bp N gene by RT-PCR. Lane M, 100 bp ladder; Lane 1, negative control; Lane 2, Infected goat lung; Lane 3, Infected goat spleen; Lane 4, Infected goat cecum.

Viral infection in the lung and spleen of PPRV infected tissue was confirmed by sandwich ELISA. Moreover, the N gene amplicon of 351 bp amplified from cDNA synthesized from infected lung and spleen confirmed viral infection (Figure 1). The viral infection was further confirmed by RT-qPCR in the infected tissues as per the protocol used in our previous study [32].

### Identification, classification, and expression analysis of lncRNA

**Figure 2.**
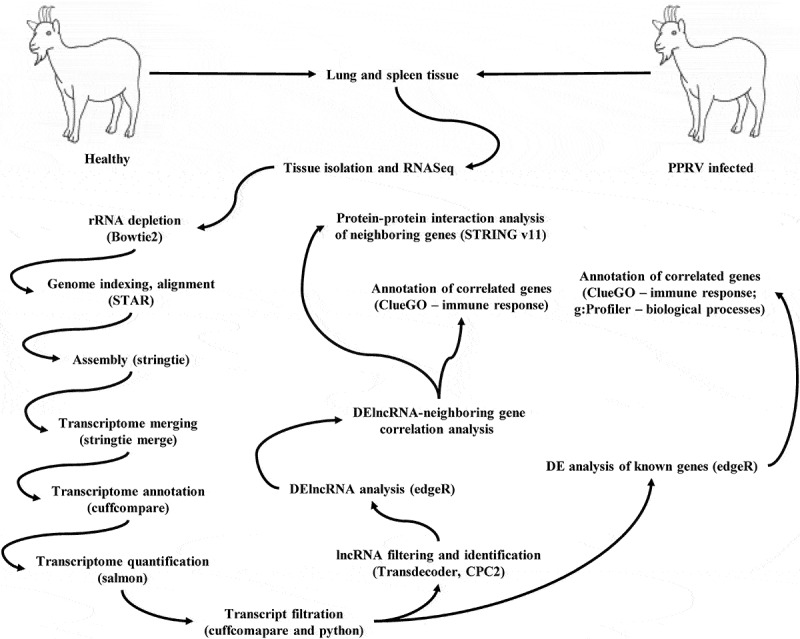
Overview of identification of lncRNAs, mRNAs, and neighboring genes of lncRNAs.

**Figure 3.**
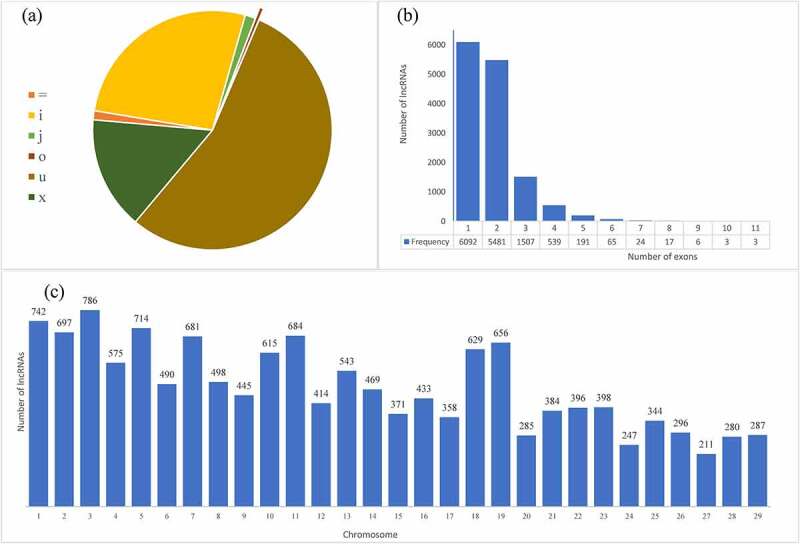
**Genomic features of lncRNAs in PPRV infected and uninfected control lung and spleen tissues of goat**. (a) Distribution of predicted lncRNAs as per class code. (b) Distribution of the number of exons in the predicted lncRNA transcripts (c) Chromosome-wise distribution of predicted lncRNA transcripts.

The lncRNA prediction pipeline is shown in Figure 2. A total of 13,928 lncRNA transcripts were identified, out of which 170 were known lncRNAs (class code “ = ”). Of the novel lncRNA transcripts, 7625 were intergenic (class code “u”), 3735 were entirely intronic (class code “i”), 2128 had exonic overlaps with the reference on the opposite strand (class code “x”), 195 were novel isoforms of known genes (class code “j”), and 75 had exons that overlapped with a reference transcript (class code “o”). The percentage-wise distribution is shown in Figure 3a and Table 1.

**Table 1.** Distribution of lncRNAs as per class code

Class code	=	i	j	o	u	x
Number of lncRNAs	170	3735	195	75	7625	2128
Percentage	1.22	26.82	1.40	0.54	54.75	15.28

Recent studies in mammals have shown that lncRNAs are shorter in length and have fewer exons than mRNAs 33, 34]. To determine whether the novel lncRNAs detected in our study have the same features, we calculated exon numbers in novel lncRNAs and neighboring mRNAs/genes. The lncRNAs ranged in length from 200 to 5000 bp and in the number of exons from 1 to 11 (Figure 3b; Table 2). The neighboring mRNAs were identified with an average length of 3019 nucleotides (nts) and median length of 2319 nts which was longer than the lncRNAs, having an average length of 768 nts and median length of 439 nts. Furthermore, the identified lncRNAs tended to have fewer exons with 1 exon being the most common (6092 lncRNAs), followed by 2 exons (5481 lncRNAs) and 11 exons as the highest number (3 lncRNAs) (Figure 3b).

**Table 2.** Summary of transcript length of lncRNAs and their host/neighboring genes as obtained from LncEvo pipeline

	Min.	1st Quartile	Median	Mean	3rd Quartile	Max.
lncRNA	200	276	439	767.7	881	12,909
Neighboring genes	70	1210	2319	3019	4250	26,406

### Differential expression and genomic distribution of lncRNAs and mRNAs

Out of 3 infected and 3 control samples, each for lung and spleen, a total of 4 samples (1 per group) had to be excluded from differential expression analysis as suspected outliers (based on hierarchical clustering and PCA plots). Further filtering was done by removing lncRNAs with an average count per million value less than 1, thus retaining 5983 and 5935 lncRNA for spleen and lung, respectively. Differential analysis of the remaining lncRNAs using edgeR revealed that 15 lncRNAs were differentially expressed (11 downregulated and 4 upregulated) with FDR < 0.05 in the PPRV infected spleen samples as compared to control. Similarly, a total of 16 lncRNAs were differentially expressed (13 downregulated and 3 upregulated) with FDR < 0.05 in the infected lung samples as compared to control. The list of DElncRNAs is given in Table 3. Two lncRNAs MSTRG.13479 and MSTRG.12371, both downregulated, were found to be common between lung and spleen samples (Supplementary figure 1). The nucleotide sequences of DElncRNAs are given in Additional file 1.

**Table 3.** List of DElncRNAs from lung and spleen

Lung tissue			Spleen tissue		
lncRNA	logFC	FDR	lncRNA	logFC	FDR
MSTRG.35687	6.770839	0.033183	MSTRG.15689	7.691874	0.0002
MSTRG.33618	6.697032	0.040448	MSTRG.21836	7.3523	0.0002
MSTRG.32946	5.204771	0.033135	MSTRG.17243	6.427333	0.016632
MSTRG.34679	−4.48949	0.028963	MSTRG.17056	5.891467	0.00898
MSTRG.4216	−5.97763	0.028963	MSTRG.25071	−3.58761	0.016419
MSTRG.9698	−6.02013	0.033795	MSTRG.40907	−5.21327	0.00898
MSTRG.30520	−6.05699	0.040448	MSTRG.30492	−5.33657	0.002035
MSTRG.16301	−6.15934	0.043806	MSTRG.41108	−5.67512	0.004402
MSTRG.38396	−6.21797	0.028963	MSTRG.12371	−5.72599	0.033155
MSTRG.1068	−6.46662	0.028963	MSTRG.40517	−5.74691	0.01448
MSTRG.10541	−6.5199	0.016315	MSTRG.27537	−6.23634	0.003329
MSTRG.312	−6.78768	0.028963	MSTRG.32595	−6.3204	0.012781
MSTRG.9699	−7.87083	0.016315	MSTRG.21354	−6.4101	3.91E-08
MSTRG.13479	−7.90389	0.004329	MSTRG.2162	−6.48377	0.001969
MSTRG.12371	−8.59352	0.001295	MSTRG.13479	−6.83393	0.000202
MSTRG.969	−8.94503	0.040448			

### Analysis of lncRNAs regulating nearby target genes

It has been reported that lncRNAs can act either in cis or in trans to regulate protein-coding gene expression [35]. We proceeded only to find the neighboring genes whose expression was highly correlated with the differentially expressed lncRNAs (Additional file 2). In the lung tissue, the DElncRNAs were found to be highly correlated with 2132 (unique) neighboring genes, whereas the DElncRNAs from spleen tissue were found to be correlated with 1624 genes. Since the functional annotation terms in ClueGO for *Capra hircus* are not well-defined and given the fact that protein functions are conserved across species, human orthologs (712 in lung and 570 in spleen) of the neighboring genes correlated with and potentially governed by the DElncRNAs were subjected to ClueGO analysis.

**Figure 4.**
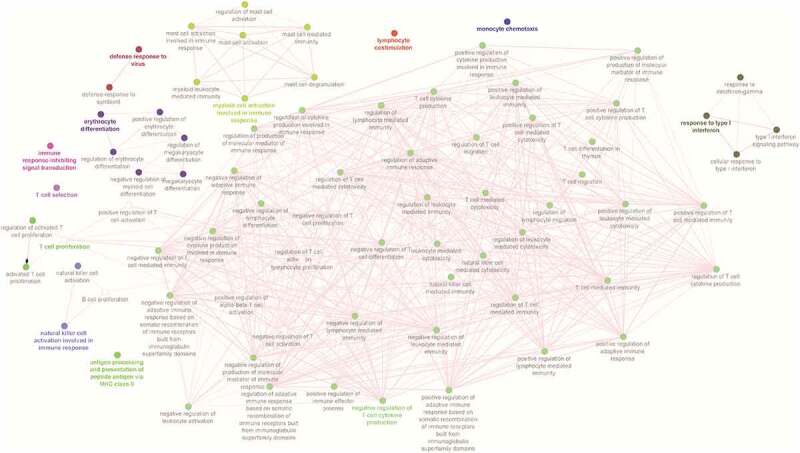
Network of immune processes enriched for neighborhood genes whose expression was found to be highly correlated with DElncRNAs in the lung.

**Figure 5.**
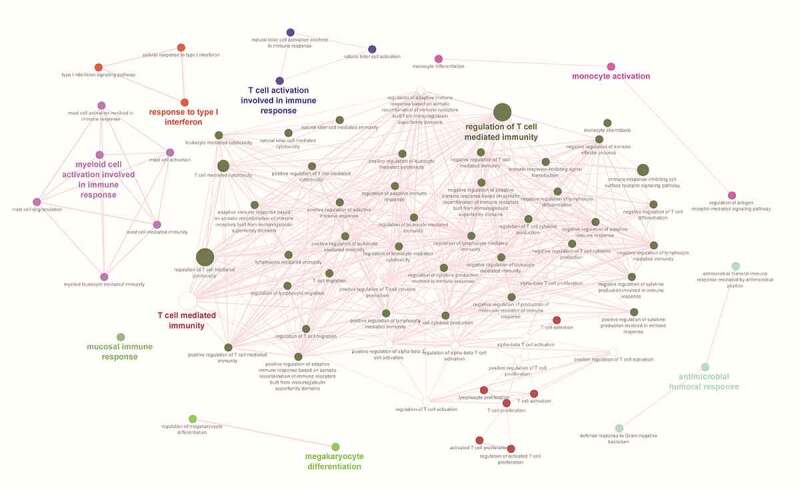
Network of immune processes enriched for neighborhood genes whose expression was found to be highly correlated with DElncRNAs in the spleen.

In the lung, the neighboring genes were found to be involved in different immune processes, and the top significant (P-value <0.05) processes were associated with T cell response (cytokine production, T cell-mediated cytotoxicity, T cell-mediated immunity), leucocyte mediated cytotoxicity, and mast cell activation. (Figure 4). In the spleen, the top significantly enriched immunological processes were associated with T cell response (cytokine production, T cell-mediated cytotoxicity, T cell-mediated immunity, T cell proliferation), NK cell activation, immune signaling (cell surface receptor signaling pathway, signal transduction), regulation of adaptive immune response, leukocyte mediated cytotoxicity, monocyte activation, and mast cell activation (Figure 5).

**Figure 6.**
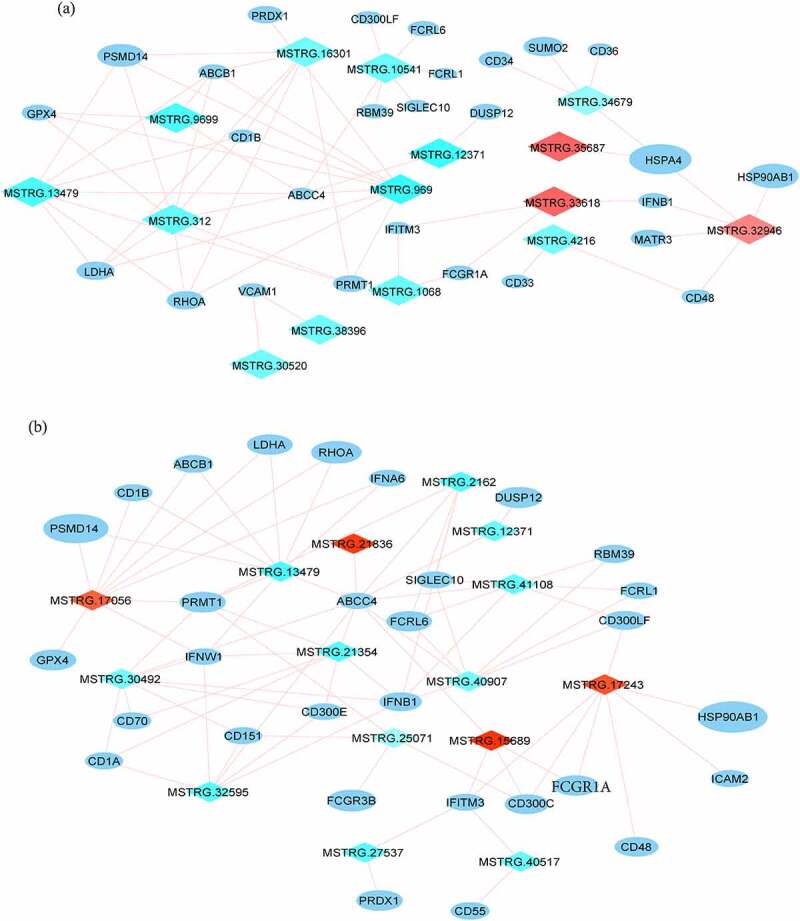
**DElncRNA-selected neighboring genes regulatory network involved in immune processes**. (a) Network of DElncRNAs (upregulated and downregulated) and the genes they regulate in the lung tissue. (b) Network of DElncRNAs (upregulated and downregulated) and the genes they regulate in the spleen tissue. DElncRNAs are represented by diamonds, with upregulated lncRNAs shown in red and downregulated lncRNAs in cyan. The neighboring genes are shown as ellipses, whose size is a function of their connectivity in protein-protein interactions (as shown in supplementary figure 2).

The ortholog genes from the spleen (570) and lung (712) were subjected to protein-protein interaction (PPI) analysis against the STRING v11 database. A total of 648 genes from the lung were found to have 2338 interactions (Supplementary figure 2A). Similarly, a total of 503 genes (nodes) from the spleen were found to have 1700 interactions (edges) in the database (Supplementary figure 2B). Since the networks were dense, a list of highly connected genes, termed hubs, was obtained using custom R scripts. In addition to genes involved in protein metabolism (UBA52, RPL6, RPL9, RPL12, RPL30, RPS13, RPL13, RPL5, RPL7, RPS14, GNB1, HSP90AB1, MRPL13 in both spleen and lung), immune-related genes like ABCB1, ABCC4, CD1B, CD300LF, CD48, DUSP12, FCGR1A, FCRL1, FCRL6, GPX4, HSP90AB1, IFITM3, IFNB1, LDHA, PRDX1, PRMT1, PSMD14, RBM39, RHOA, and SIGLEC10 were among the hub genes whose expression was highly correlated with the DElncRNAs in both lung and spleen. In addition to these, genes such as ATP5A1, CD33, CD34, CD36, HSPA4, MATR3, SUMO2, and VCAM1 were identified as key genes in the PPI network of the lung tissue, whereas FCGR3B, PARK2, CD300C, CD55, CD151, CD1A, CD70, ICAM2, CD300E, IFNA6, and IFNW1 were identified in the spleen tissue. The PPI networks and the interactions between immune-related genes and DElncRNAs were visualized in Cytoscape. As shown in the DElncRNA-immune genes network (Figure 6), except MSTRG.12371, all other DElncRNAs in the spleen tissue are connected with more than one immune-related gene and vice versa, thus potentially modulating gene expression through multiple interactions. A similar network was seen in the lung tissue where multiple DElncRNAs interact with different genes to coordinate their expression, except VCAM whose expression is modulated by MSTRG.38396 and MSTRG.30520 as the trio forms a network that is separate from other genes.

### Gene Ontology (GO) analysis of DEmRNAs

**Figure 7.**
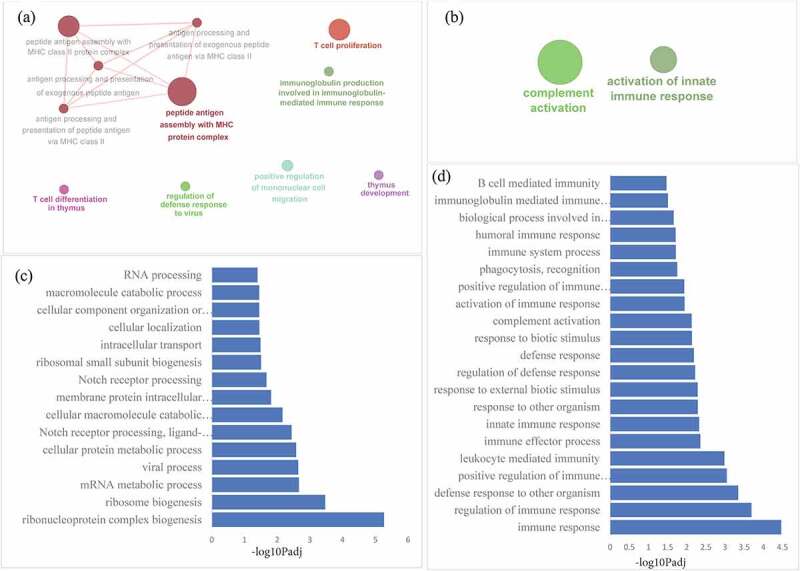
**Gene Ontology analysis of differentially expressed genes**. Functional enrichment analysis of DEmRNAs in lung and spleen showing immune pathways enriched in lung (a), immune pathways enriched in spleen (b), biological processes enriched in lung (c), and biological processes enriched in spleen (d) of PPRV infected goats.

A total of 290 known genes (mRNAs) in the lung and 67 genes in the spleen were found to be differentially expressed (FDR < 0.05) (Additional file 3; Supplementary Figure 3). Human orthologs of differentially expressed genes were subjected to gene enrichment analysis for immune pathways using ClueGO and biological processes using g:Profiler. In the lung, top enriched immune-related terms included those involved in antigen processing and presentation of exogenous peptide antigen, MHC class II activity, thymus development, and T-cell proliferation and differentiation, whereas top enriched biological processes included those involved in protein metabolism, ribosome biogenesis, viral processes, and Notch receptor signaling. In the spleen, top enriched immune-related terms included complement activation and activation of the innate immune response, whereas top enriched biological processes included those involved in positive regulation of immune response, leukocyte activation, innate immune response, complement activation, pathogen recognition, and phagocytosis, and B cell-mediated immunity. Network of enriched immune pathways and list of biological processes (plotted against negative log_10_ transformed adjusted p-value) each in lung and spleen are provided in Figure 7.

## Discussion

PPRV infection is characterized by high fever, high morbidity, and high mortality and results in tremendous economic losses. Although much advance has been achieved in PPRV biology and anti-PPRV immune response, the mechanism by which PPRV causes the fatal disease PPR is still not fully understood. Recently, many studies have suggested that host-encoded lncRNAs play key roles in regulating immune response against viral infection [21, 22, 36, 37, 38]. However, the role of lncRNAs in PPRV infection remains unclear.

In the present study, we predicted and characterized novel lncRNAs whose expression levels were altered after PPRV infection in the lung and spleen of goats. The expression of lncRNAs was found to be tissue-specific. The majority of lncRNAs in the lung and spleen belong to the intergenic (“u”) type, followed by intronic (“i”) lncRNAs and exonic lncRNAs on the opposite strand. Even though no specific sequence or structural feature defines lncRNAs, it has been reported that lncRNAs are less enriched in expression, shorter in length, and have fewer exons in comparison to protein-coding genes [25,39]. In agreement with the above findings, we have also detected lncRNAs with shorter lengths and fewer exons. These similarities supported that the potential lncRNAs identified in this study were reliable. Moreover, a total of 16 and 15 lncRNAs were dysregulated in the lung and spleen, respectively, and the downregulated lncRNAs outnumbered upregulated lncRNAs in both tissues. We also observed that DElncRNA transcripts were not evenly distributed across all the chromosomes (Figure 3c).

One more aspect of lncRNAs is that they play a regulatory role by interacting with protein-coding genes through cis- and trans-regulatory mechanisms [35]. The DElncRNAs showed correlated expression with a large number of neighboring genes, whose expression they potentially regulate during PPRV infection. Functional analysis of these highly correlated neighboring genes suggests that the DElncRNAs might have a role to play in regulating immune processes. The top-most immune processes for which these genes were enriched include T-cell mediated immunity, natural killer cell activation, and antigen processing and presentation, suggesting both innate and adaptive immune responses are regulated by lncRNAs under PPRV infection.

In an attempt to understand more about lncRNA and the interaction of their neighboring genes, we constructed a lncRNA–gene interconnecting network. We found that the ATP-binding cassette (ABC) transporters ABCB1 and ABCC4 potentially interact with multiple lncRNAs. While, in the lung, all the lncRNAs interacting with ABCB1 and ABCC4 are downregulated, in the spleen, ABCB1 expression is correlated with upregulated MSTRG.17056 and downregulated MSTRG.13479, and ABCC4 is correlated with upregulated MSTRG.21836 and at least nine down-regulated lncRNAs. ABCB1 and ABCC4 have been reported to be involved in dendritic cell migration toward the draining lymph nodes in humans as well as mice [40,41].

Various interferons were found to have a correlated expression with the DElncRNAs. IFNB1 in both spleen and lung showed correlated expression with multiple lncRNAs. In the lung, the potential interacting lncRNAs were MSTRG.33618 and MSTRG.32946 (both upregulated), whereas, in the spleen, all the six lncRNAs that interact with IFNB1 were downregulated. Moreover, in the spleen, interferons IFNA6 and IFNW1 also were a part of the DElncRNA-neighboring gene network. Interferons are a part of the innate immune system against viral infection and comprise a class of molecules upregulated in viral infections in most animals [42]. IFNW1 is mainly expressed in virus-infected leucocytes, binds to the same class I IFN receptor complex as IFN-alpha and IFN-beta but differs antigenically, and has potent antiviral activity against various DNA and RNA viruses [43,44]. We also observed that Interferon-induced transmembrane protein 3 (IFITM3) expression was correlated with upregulated MSTRG.33618 and downregulated MSTRG.1068 in the lung, and 2 upregulated (MSTRG.15689 and MSTRG.17243) and 2 downregulated (MSTRG.27537 and MSTRG.40517) DElncRNAs in the spleen. IFITM3 belongs to IFITM class of immune effectors that act as the first line of cellular defense against viruses and prevent viral entry into the cells [45].

In our study, we found that expression of various members of Fc receptor-like (FCRL) and Fc gamma receptor families correlated with the expression of DElncRNAs – FCRL1, FCRL6, and FCGR1A were found in the lung as well as spleen networks, whereas FCGR3B was found only in the spleen. Members of the FCRL family, expressed by B-cells, are a class of immunoregulatory proteins that modulate adaptive and innate signaling pathways and immune cell development [46,47]. Similarly, Fc gamma receptors play diverse roles in phagocytosis, antigen presentation, and antibody-dependent cell killing [48]. FCGR1A is also a robust biomarker for viral and bacterial respiratory infections [49].

Among the cell surface proteins that had correlated expression with DElncRNAs, SIGLEC10 is connected to downregulated MSTRG.10541 in the lung and downregulated MSTRG.41108, MSTRG.2162, and MSTRG.40907 in the spleen. It acts as an inhibitory immune receptor and regulates B-cell antigen receptor signaling and NK signaling [50,51]. Vascular cell adhesion molecule 1 (VCAM1) and its correlated DElncRNAs MSTRG.38396 and MSTRG.30520 form a separate cluster in the lung tissue. VCAM1 is an endothelial cell-derived member of the VCAM family of proteins involved in regulating various inflammatory processes [52].

Functional/enrichment analysis of DElncRNAs revealed that enriched immune pathways and biological processes were more or less similar to those observed in enrichment analysis of neighboring genes. To the best of our knowledge, this is the first report that highlights the identification and expression profiles of lncRNAs in the lung and spleen of goats after PPRV infection. These findings support our hypothesis that DElncRNAs and DEmRNAs raise a coordinated immune response in the lung and spleen of PPRV infected goats. The results suggest that PPRV infection might regulate the host immune response by inducing lncRNAs that act as regulatory elements in the host immune systems. Furthermore, the identified lncRNAs can contribute to the annotation of the goat genome. However, further efforts are required to confirm the present findings.

## Materials and methods

### Ethics statement and animal experiment

The vaccine potency testing experiments were carried out at ICAR-Indian Veterinary Research Institute Mukteshwar Campus as per the guidelines of Indian Pharmacopoeia – 2014 (page.no: 3626). All experimental protocols were approved by the Institutional Animal Ethics Committee (IVRI-IAEC). In the present study, healthy goats (1 yr. old), tested negative for the presence of PPRV antibody by competitive ELISA [53] and serum neutralization test (SNT) [54] were infected with a virulent PPRV strain (Izatnagar/94 – lineage IV, KR140086.1) [32] using a 10% splenic suspension of the virulent virus [4 ml suspension, 2 ml each at two different sites subcutaneously) as mentioned in [31]. The infected animals were monitored diurnally for rectal temperature and secretion from natural orifices and feeding habits throughout the experimental period. During the infection period, the infected animals developed symptoms characteristics of PPRV. The infected animals in which temperature dropped subnormal were sacrificed at 10 days post-infection. Tissue samples – lung and spleen were collected from PPRV infected goats (n = 3]. PPRV infection in the lung and spleen was confirmed by RT-PCR, qRT-PCR, and sandwich ELISA [55]. Healthy tissues and serum of three apparently healthy goats were collected from the nearby slaughterhouse and screened for the absence of PPRV antigen by sandwich ELISA and PPRV antibodies by competitive ELISA, respectively.

### RNA extraction and sequencing

Total RNA from each of the collected samples (lung and spleen; n = 3 each) was isolated using the RNeasy Mini kit (Qiagen GmbH, Germany) according to the manufacturer’s protocol. The integrity and quantity of isolated RNA were assessed on a Bioanalyzer (Agilent Technologies, Inc). The library was prepared using NEBNext Ultra RNA Library Prep Kit for Illumina (New England Biolabs Inc.) following the manufacturer’s protocol. Approximately, 100ng of RNA from each sample was used for RNA library preparation. The quality of the libraries was assessed on Bioanalyzer. Libraries were quantified using a Qubit 2.0 Fluorometer (Life Technologies) and by qPCR. The library (1.3 ml, 1.8pM) was denatured, diluted, and loaded onto a flow cell for sequencing. cDNA library preparation and Illumina Sequencing (NextSeq 500; 100 bp paired-end) were performed. Various parameters of the RNA-Seq dataset have been presented in Supplementary Table 1.

### Assembling reads and identification of lncRNAs

Raw RNA-Seq reads were processed as per the LncEvo pipeline [56] with modifications. LncEvo is an all-in-one Nextflow based pipeline for transcriptome assembly and lncRNA identification, which takes paired FASTQ reads and genome assembly IDs as the input and uses customized scripts to essentially automate the process. Briefly, the latest goat genome (ARS1.dna.toplevel.fa; release 104) and corresponding annotation files were downloaded. Bowtie2 [57] was used to align the input FASTQ files to known goat rRNA sequences and only unmapped reads were retained. The reference genome was indexed and the retained reads were then mapped against the genome using STAR v2.7.3a with default parameters [58]. Stringtie v2.1.1 [59] was used for ab initio transcriptome assembly from the BAM files generated in the previous step, and a custom transcriptome (GTF) was obtained for each sample. Individual GTF files were merged into a single transcriptome that was compared against the reference annotation using Cuffcompare [60] to remove potential errors (class codes c, e, p, s). FASTA sequences of the filtered transcriptome were extracted using gffread v0.11.7 [61], and subsequently indexed in and used for transcript-level quantification in Salmon v0.12.0 [62].

After quantification, the transcriptome was again annotated against the reference annotation using Cuffcompare, followed by lncRNA filtering steps viz., i) transcripts with class codes “ = ”, “j”, or “o” were removed if the reference gene isn’t already classified as lncRNA; ii) transcripts shorter than 200 bp length were discarded; iii) strand-specific transcripts (-S option) and those with ORF that have a minimum protein length of 100 (-m 100) as identified by TransDecoder (https://github.com/TransDecoder/TransDecoder) were discarded; iv) Transcripts classified as encoding by Coding Potential Calculator (CPC) 2 standalone version [CPC2.0 beta; 63] were discarded.

### Identification of DElncRNAs

The remaining transcripts identified as potential lncRNAs were retained for differential expression analysis. A count (transcripts per million; TPM) matrix was generated using the tximport v1.20.0 package in RStudio. Read counts were used to plot the hierarchical clustering of the samples, which revealed that 2 samples each in spleen and lung (and one each from infected and control groups) tended to cluster away from the rest of the samples. This was confirmed by constructing a PCA plot for all the samples. The erratic samples were excluded from downstream analysis. The differential expression analysis separately for lung and spleen of goat (n = 2 each) between the healthy and infected tissues was performed using edgeR v3.34.1 [64] after discarding transcripts with low expression levels.

### Target genes and functional prediction of DElncRNAs and DEmRNAs

To explore the functions of lncRNAs, we predicted the cis-regulated target genes of the DElncRNAs located on the same chromosome. We searched for genes that are either close to or overlapping with DElncRNAs (within a window of 1 Mb upstream or downstream of DElncRNAs) using the “bedtools window” tool v2.27.1 [65]. The neighboring genes whose expression was correlated with the expression of DElncRNAs (|correlation| > 0.8) were retrieved for functional enrichment analysis. Immune system processes in ClueGO v2.5.8 [66] of Cytoscape v3.8.2 [67] were selected to perform the enrichment analyses of these genes potentially regulated by DElncRNAs. In addition to DElncRNAs and their neighboring genes, functional annotation of DEmRNAs was also performed against immune pathways in ClueGO and Biological Processes in g:Profiler [68].

## Supplementary Material

Supplemental Material

## Data Availability

The datasets for this study were deposited in SRA (SRP194369).

## Abbreviations

PPRV: Peste des petits ruminants virus; lncRNA: long non-coding RNA; DElncRNA: differentially expressed lncRNA; DEmRNA: differentially expressed mRNA; NRAV: negative regulator of antiviral response; NLRP3: NOD-, LRR- and pyrin domain-containing protein 3; THUMPD3-AS1: THUMPD3 Antisense RNA 1; NEAT1: Nuclear Paraspeckle Assembly Transcript 1; IL: Interleukin; SIGLEC10: Sialic Acid Binding Ig Like Lectin 10; RPs: various Ribosomal Proteins; CDs: clusters of differentiation.
