# Supplementary figures and images for: Differential expression of long non-coding RNAs under Peste des petits ruminants virus (PPRV) infection in goats
Source: Virulence. 2022 Feb 6;13(1):310–22. doi: 10.1080/21505594.2022.2026564 (PMC8824212; doi:10.1080/21505594.2022.2026564)

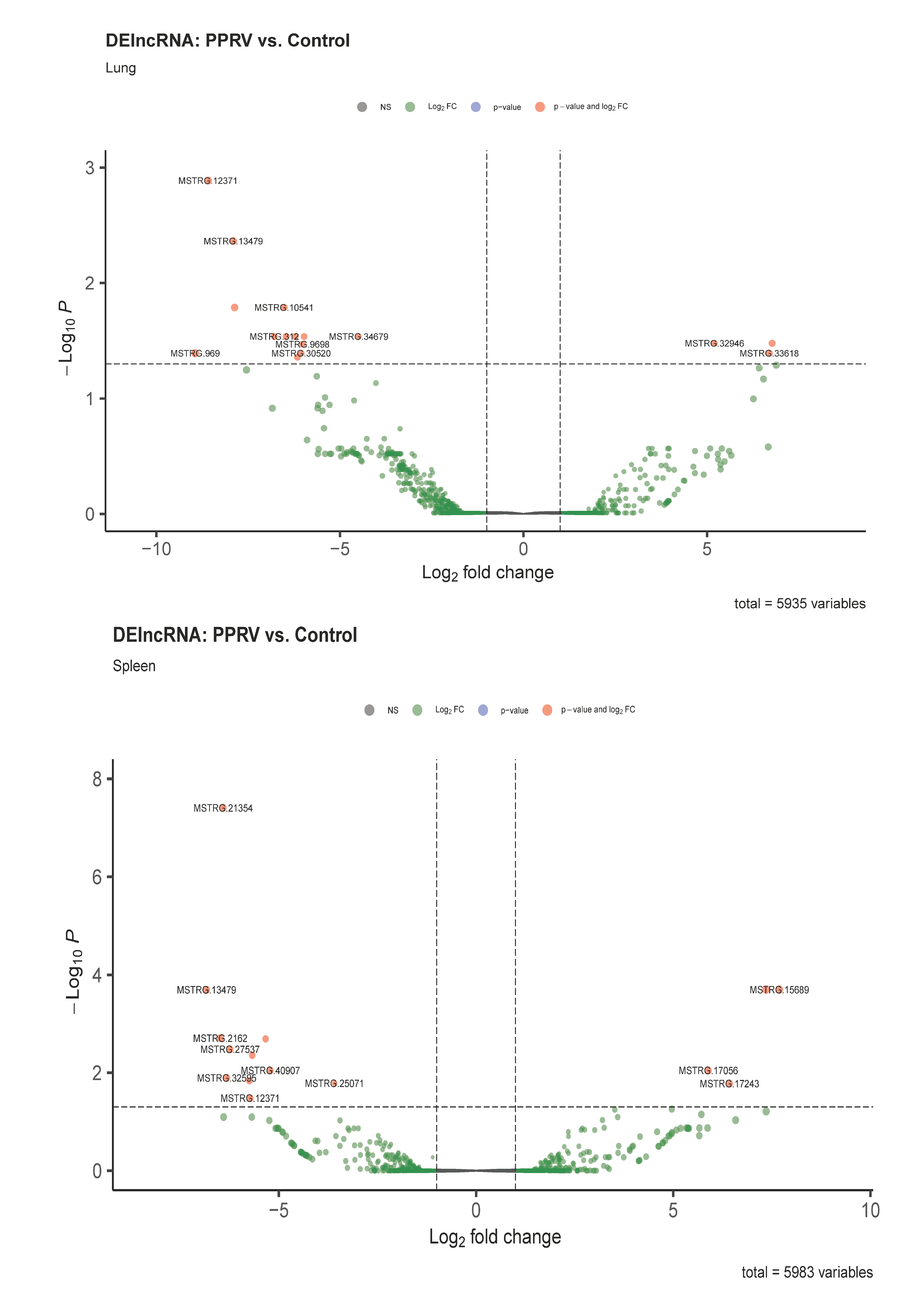

Supplement: Supplemental Material [file KVIR_A_2026564_SM6977.zip › supplementary/Supplementary 1.tiff]

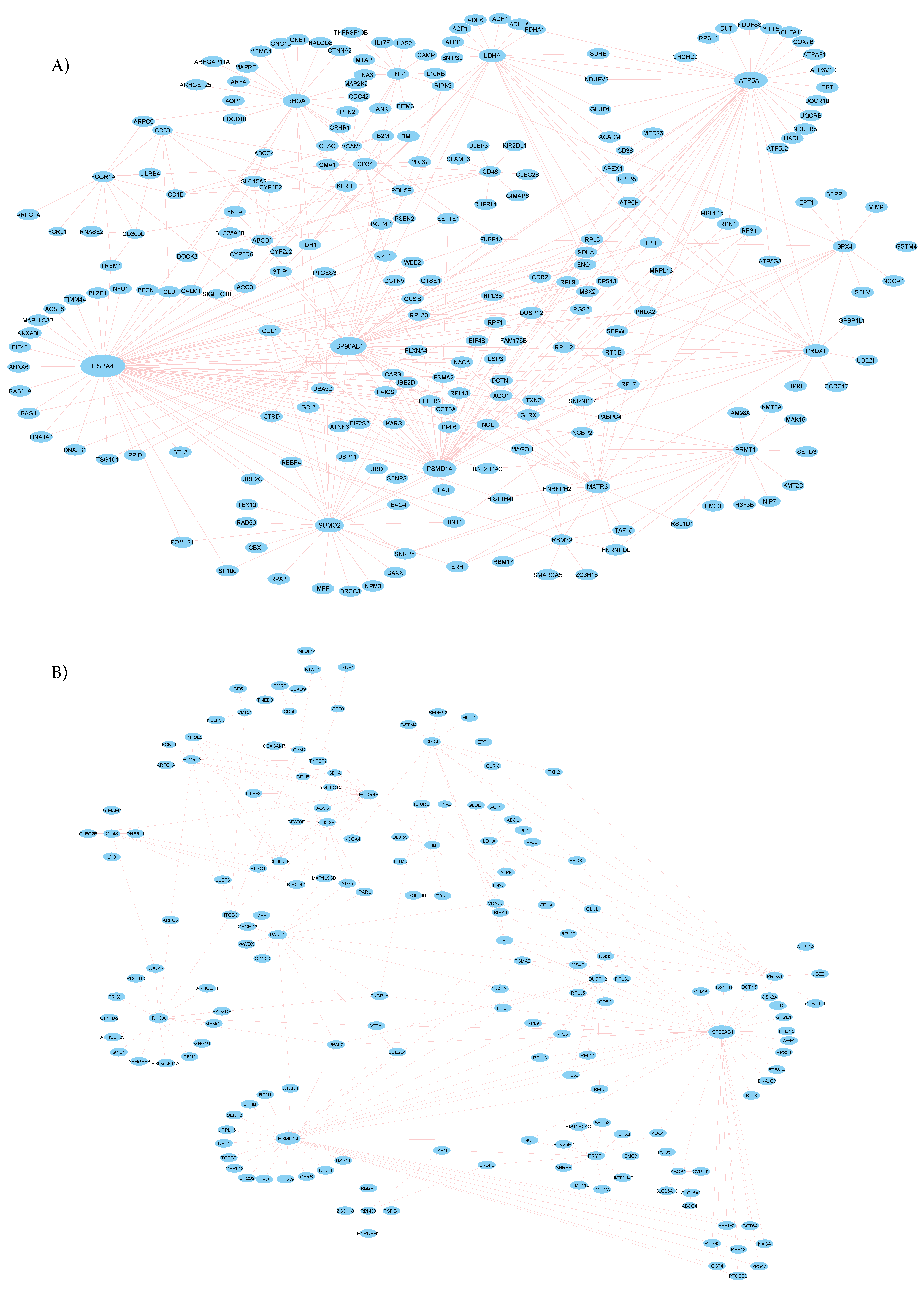

Supplement: Supplemental Material [file KVIR_A_2026564_SM6977.zip › supplementary/Supplementary 2.tiff]

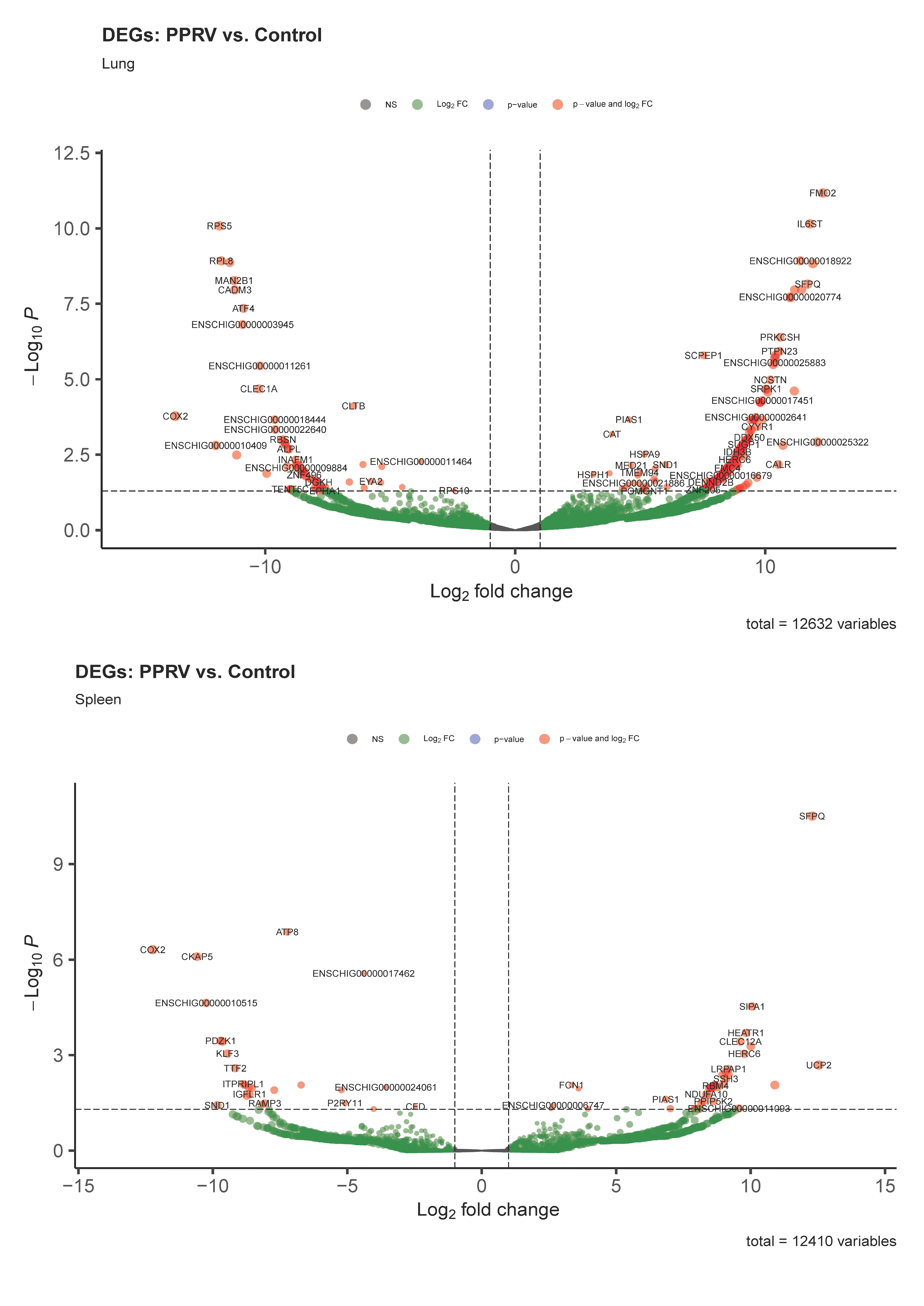

Supplement: Supplemental Material [file KVIR_A_2026564_SM6977.zip › supplementary/Supplementary 3.tiff]
